# Distinguishing citrus varieties based on genetic and compositional analyses

**DOI:** 10.1371/journal.pone.0267007

**Published:** 2022-04-18

**Authors:** Rui Min Vivian Goh, Aileen Pua, Francois Luro, Kim Huey Ee, Yunle Huang, Elodie Marchi, Shao Quan Liu, Benjamin Lassabliere, Bin Yu

**Affiliations:** 1 Department of Food Science and Technology, National University of Singapore, Singapore, Singapore; 2 Mane SEA PTE LTD, Singapore, Singapore; 3 UMR AGAP Institut, CIRAD, INRAE, Institut Agro, Univ Montpellier, San Giuliano, France; Universidad Autonoma de Chihuahua, MEXICO

## Abstract

Simple sequence repeats (SSR) markers and secondary metabolite composition were used in combination to study seven varieties of citrus for the first time. With reference to established accessions of citrus, two of the varieties (Chanh Giay and Ma Nao Pan) were predicted to be Mexican key limes, while three were mandarin hybrids (Nagpur, Pontianak and Dalandan) and the remaining two (Qicheng and Mosambi) were related to the sweet orange. Notably, Dalandan was genetically more like a mandarin despite often referred to as an orange locally, whereas Mosambi was more likely to be a sweet orange hybrid although it has also been called a sweet lime due to its green peel and small size. Several key secondary metabolites such as polymethoxyflavones (sinensetin, tangeretin etc.), furanocoumarins (bergapten, citropten etc.) and volatiles (citronellol, α-sinensal etc.) were identified to be potential biomarkers for separation of citrus species. However, despite having similar genetic profiles, variations in the volatile profile of the two limes were observed; similarly, there were differences in the secondary metabolite profiles of the three mandarin hybrids despite having a common ancestral parent, highlighting the usefulness of genetic and compositional analyses in combination for revealing both origins and flavour profiles especially in citrus hybrids. This knowledge would be crucial for variety screening and selection for use in flavour or fragrance creation and application.

## 1. Introduction

The *Citrus* genus is a complicated genus comprising of many hybrid fruits. Beginning from just ten progenitor species across Asia and Australia, there now exists hundreds of citrus species all over the world [1, 2]. Most of the citrus species widely propagated, sold and consumed worldwide are a descendant of these progenitor species, including common citrus fruits such as orange (*Citrus sinensis*) and lemon (*Citrus limon*) [3]. Studies of existing and new citrus hybrids with exotic or palatable taste and aroma properties have been of sustained interest especially in the food, flavour and perfumery industries [4–6]. Although citrus encompasses some of the most commonly consumed fruits in the world, some niche varieties are only consumed locally and are often unknown to the rest of the world despite having unique flavours [7]. The Mosambi, originating from India, is one such fruit. Mosambi is a common name shared by two reported hybrid citrus fruits in India: *Citrus sinensis* Osbeck (sweet orange), which is described to have yellow peel with low acidity; and *Citrus limetta*, a citron *(Citrus medica*) and sour orange (*Citrus aurantium*) hybrid which has green peel and is often also referred to as sweet lime or sweet lemon [7–9].

Investigations into the genetic origins of these hybrids are often conducted using genetic analyses focusing on specific markers known to be unique to each type of parental species and can build on the results of analytical studies for further confirmation of the findings [10]. However, correlating these genetic markers to the physicochemical properties of these hybrids is challenging due to phenotypic changes that can arise from agricultural differences, postharvest conditions or spontaneous somatic mutations, resulting in variations that are not explainable by genetic makeup [11]. Therefore, while genetic analysis can reveal the origins of these hybrid citrus fruits, it may not reflect the differences in flavour profiles expressed in citrus hybrids.

Like their genetic profiles, flavour profiles of citrus are also unique to each variety of citrus. Compositional analyses of citrus are thus equally essential to study their final compositions, which are more relevant for their applications, such as in flavour and fragrance creation or pharmaceutical products [12, 13]. Secondary metabolites are often the target of these analyses, being recognised as key marker compounds in most citrus fruits, including characteristic non-volatile compounds such as sinensetin and limonin, and key aroma compounds like citronellol [12, 14, 15]. In recent years, high-resolution detectors have been increasingly employed for the analysis of secondary metabolites in natural food products, including citrus, to overcome challenges such as trace abundances or chromatographic separation [12, 16]. These flavour profiles can give insights into the similarities between each variety, drawing possible links between different accessions of citrus [14]. This knowledge will greatly help in establishing the unique flavour profile of citrus hybrids and aid in the creation of these flavours with reference to other relative species.

Thus far, most citrus studies have applied genetic and chemical analyses independently in their approaches to better understand these complex fruits. However, an integration of genetic, compositional and statistical methods would contribute to a more holistic understanding of these hybrid fruits that possess a complex metabolome. Additionally, citrus fruits with similar genetic profiles may have varying secondary metabolite compositions and therefore have different flavour properties, while other hybrids that appear to be alike based on their secondary metabolite profiles can have differing genetic profiles, pointing to having different origins or ancestral species. This was explored in this study with seven varieties of citrus grown in Asia. These varieties include one mandarin (Pontianak), three oranges (Qicheng, Nagpur, Dalandan), two limes (Chanh Giay and Ma Nao Pan) and Mosambi which had green peel. Simple sequence repeats (SSR) markers were used for genotyping and the species were visualised on a neighbour joining (NJ) tree. Concurrently, the composition of secondary metabolites in the peel (non-volatiles and volatiles) and in the juice (non-volatiles) were analysed using liquid chromatography (LC) with a quadrupole-time-of-flight (QTOF) detector, and gas chromatography (GC) coupled with mass spectrometry (MS) and a flame ionisation detector (FID). The genetic and secondary metabolite profiles were then compared using statistical analysis and the similarities between the genotypes and phenotypes were evaluated.

## 2. Materials and methods

### 2.1. Chemical standards

Analytical grade standards (at least 95% purity) were used for quantification. 7-Methoxycoumarin, hesperidin, neohesperidin, scoparone were obtained from J&K Scientific Ltd. (Beijing, China). Bergamotine, bergapten, citropten, didymin, eriocitrin, hyperoside, isomangiferin, isoquercetin, isovitexin, limonin, luteolin-6-glucoside, mangiferin, narirutin, nobiletin, nomilin, orientin, rhoifolin, rutin, sinensetin, tangeretin, vicenin-1, vicenin-2 and vitexin were obtained from Wuhan ChemFaces Biochemical Co., Ltd. (Hubei, China). Chrysoeriol-7-glucoside, homoeriodictyol-7-glucoside, isorhamnetin, isorhamnetin-3-glucoside, isorhamnetin-3-neohesperidoside, isorhamnetin-3-rutinoside, isorhoifolin, isoscoparin, kaempferol-3-neohesperidoside, kaempferol-3-rutinoside, obacunone, prunin and quercetin-3-neohesperidoside were obtained from FineTech Industry Ltd. (Hubei, China).

### 2.2. Sample selection

Seven varieties of citrus were used: Mosambi (average weight 184 g, obtained from India, June 2019), Chanh Giay (199 g, Vietnam, July 2019), Ma Nao Pan (171 g, Thailand, June 2019), Qicheng (279 g, China, January 2019), Pontianak (103 g, Indonesia, November 2018), Dalandan (77 g, Philippines, November 2018) and Nagpur (104 g, India, November 2018). At commercial maturity, the Mosambi, Chanh Giay and Ma Nao Pan used in this study had green-coloured peel, Dalandan had pale green peel and Qicheng, Pontianak and Nagpur had orange peel. The peels were carefully separated from the albedo layer and immediately used for the extraction of volatile and non-volatile compounds. Juice was then obtained from the fruits by manual compression for extraction of non-volatile compounds.

### 2.3. Genetic analysis

Deoxyribonucleic acid (DNA) extraction from the leaves of the seven varieties was performed using the Qiagen “DNeasy” kit (Oregon, USA). In addition to the seven citrus varieties, 12 reference citrus varieties were also included for genetic analysis. These 12 additional citrus accessions originated from pathogen-free plants of the Citrus Biological Resource Centre (BRC Citrus, INRAE-CIRAD, NFS96-900) based in San Guiliano (Corsica, France) and are listed in S1 Table [17].

Genotyping was performed using 16 SSR markers (S2(a) and S2(b) Table). All of the markers were positioned on the reference genetic map and are distributed through the genome with a representation of 8 of the 9 chromosomes [18]. Amplifications were performed in a thermocycler (PTC 200, MJ Research, Massachusetts, USA) using 10 ng of DNA, 0.5 μM of each primer and 0.8 unit of *Taq* polymerase (Goldstar, Eurogentenc, Liège, Belgium). The annealing temperature was fixed for all primer pairs at 55°C. Separation of alleles was performed by electrophoresis using a SG-200-02 electrophoresis system (C.B.S. Scientific Company, California, USA) on a 6% polyacrylamide sequencing gel (acrylamide: bis-acrylamide, 19:1) (SERVA, Heidelberg, Germany), containing 7 M urea in 0.5x TBE buffer at 80 W for 2 h. Three microlitres of PCR product were mixed to an equal volume of loading buffer containing 95% formamide, 0.25% bromophenol blue and 0.25% xylen cyanol, and 10 mM of EDTA. This mixture was heated for 5 min at 94°C to denature the DNA before loading. Gels were stained with silver nitrate following the protocol detailed by Chalhoub et al. (1997) [19]. The analysis was repeated twice to eliminate false positive identifications.

DARwin software (V6) (CIRAD, Paris, France) was used to analyse the genetic relationships between the different varieties using the weighted NJ method, based on the ‘simple matching’ similarity index, which took into account the percentage of common alleles between two citrus samples divided by the total number of observed alleles [20]. Tree construction method used the trees inferred from the bootstrapped dissimilarities to assess the uncertainty of the tree structure. Concurrently, a bootstrap value was given to each edge that indicates the occurrence frequency of this edge in the bootstrapped trees. A factor analysis was also constructed based on genetic distances between each citrus accession using the DARwin software.

### 2.4. LC-QTOF/MS analysis

For LC-QTOF/MS (1290 Infinity II system with a 6550 iFunnel quadrupole time-of-flight detector (Agilent Technologies, California, USA)) analysis, 100.00 g of peels was extracted using 200.0 mL of LC-MS grade methanol (VWR, Pennsylvania, USA). After three hours, 40.0 g of anhydrous sodium sulfate (VWR, Pennsylvania, USA) was added to remove water, followed by filtering to remove the salt and peel. Lastly, concentration was performed with a rotary evaporator (Buchi, Flawil, Switzerland). Non-volatile compounds in the juice were extracted using 20.0 mL of methanol for every 10.0 mL of juice. Three biological extractions were performed for each citrus variety. The run parameters were: 40°C column temperature, 1 μL injection volume, and 0.1% formic acid (Merck, Darmstadt, Germany) in LC-MS grade water (Fisher Scientific Co., New Jersey, USA) and acetonitrile (ACN) (Fisher Scientific Co., New Jersey, USA) were used as mobile phases. The elution gradient was 0–2 min 5% ACN, 2–12 min 5–25% ACN, 12–22 min 25–95% ACN, and 22-25min 95% ACN before equilibrating back to 5% ACN for 5 min.

LC-QTOF/MS and data analysis parameters for the “All-ion” MSMS acquisition mode and Quantitative Analysis (version B.10.1) (Agilent Technologies, California, USA) were adapted from Goh et al. 2021 [21]. A calibration curve was then made for the detected compounds, with at least five points within the linear range for each compound. All runs were performed in triplicates, with pooled samples inserted periodically to ensure consistency of the instrument response. Principal component analysis (PCA) biplots of the analytical data were visualised using RStudio (Version 1.3.1093) and accompanying packages (ggbiplot).

### 2.5. GC-MS/FID analysis

Volatile compounds in the peel were extracted using the same method as non-volatiles, except dichloromethane (VWR, Pennsylvania, USA) was used as the extraction solvent instead of methanol. The chromatographic and spectrometric parameters were adapted from Goh et al. 2019 and operated on a 7890B GC system coupled with a FID and the 5977B mass selective detector (all from Agilent Technologies, California, USA) [22]. For each analysis, a 1 μL splitless injection was used, and 2-octanol (VWR, Pennsylvania, USA) was used as an internal standard. Spectra of compounds detected were matched against an in-house library and the NIST 14 library, and linear retention indices were determined using C7-40 alkane standards (Supelco, Pennsylvania, USA). Data analysis was carried out on the MSD Chemstation software (ver. F.01.03.2357) (Agilent Technologies, California, USA). PCA biplots were constructed using concentrations of key volatiles as well as volatiles categorised by functional group using RStudio (Version 1.3.1093) and accompanying packages (ggbiplot).

## 3. Results and discussion

Over the years, there has been increased recognition and use of simple sequence repeats (SSR) markers for citrus studies due to its usefulness in unveiling the complexities of the citrus genetic diversity. The high polymorphic and codominant traits of these SSR markers allow for accurate determination of phylogenetic relationships within a taxonomic class, which makes it particularly suitable for this highly crossbred genus [23, 24]. Many studies have utilised SSR markers to propose the origins of citrus hybrids and probable backcrosses, using a structure centred on the three recognised ancestral citrus species (citron, mandarin and pomelo) [24].

While local hybrids such as the Dalandan and Nagpur both share a similar species name (*Citrus reticulata*), the differences in their physical characteristics alone suggest different genetic origins which could be a result of different extents of backcrossing. Instead, Nagpur was more similar to Pontianak (*Citrus nobilis* or ‘tangor’) in terms of shape, colour and mass, despite belonging to a different species. Mosambi was physically similar to Chanh Giay and Ma Nao Pan, although the former is recognised as a sweet lime (*Citrus limetta*) or orange (*Citrus sinensis*), and the latter two are limes. An analysis using SSR markers was thus carried out to explore the genetic diversity of these varieties.

### 3.1. Analysis of SSR markers

**Fig 1:**
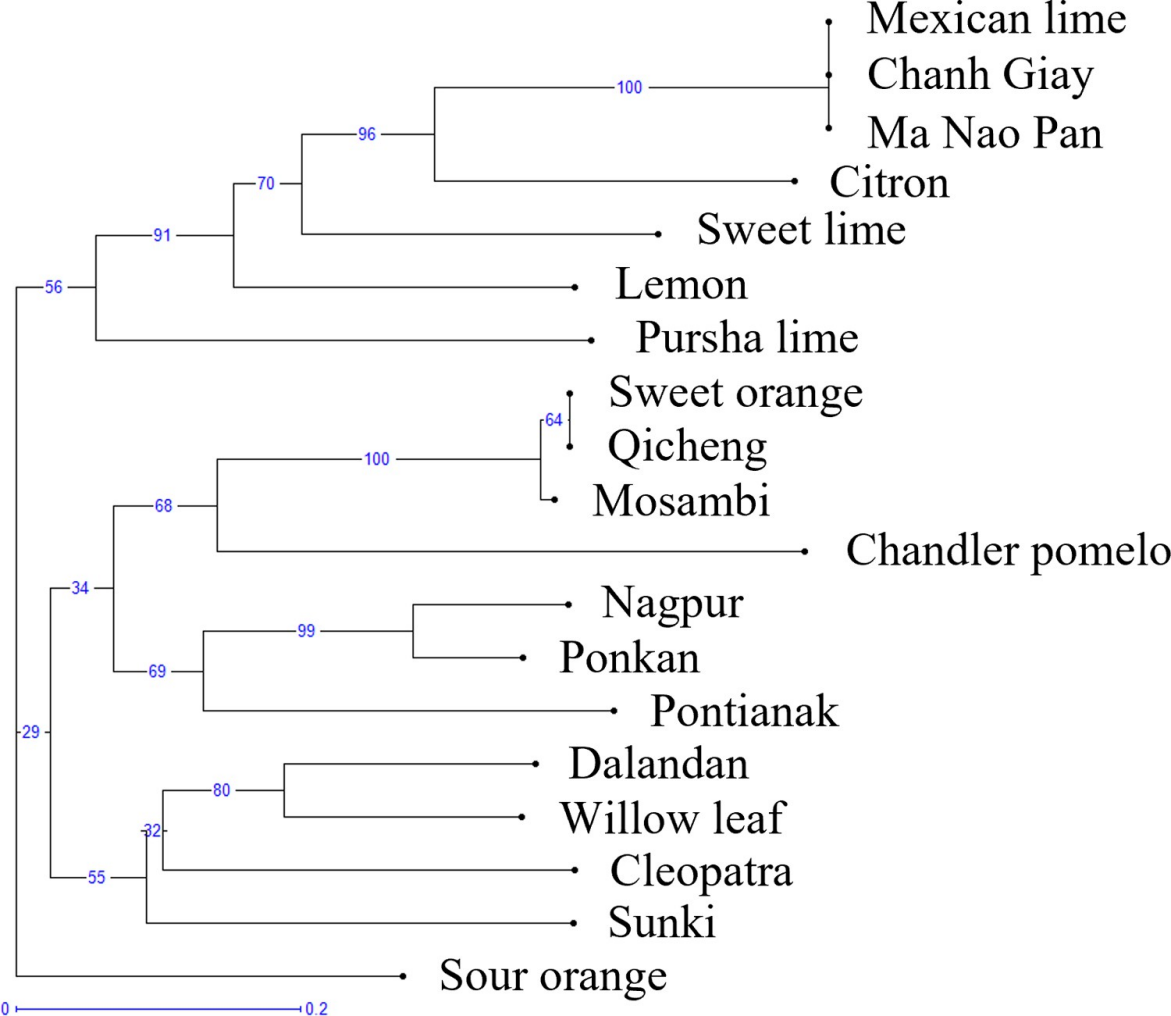
Neighbour-joining (NJ) tree for the seven Asian citrus varieties and 12 reference citrus varieties (S1 Table) established with the allelic data of 16 SSR markers (S2(a) Table). Bootstrap values are located on each branch.

The relationships between different varieties of citrus are illustrated on the NJ tree, where the length of branches that connect all the genotypes are proportional to genetic distances (Fig 1). Each ancestral species (citron, mandarin and pomelo) represented a pole of diversity and the interspecific hybrids were observed to be distributed closely to their ancestral species. Generally, the placement of the reference citruses was in agreement with the hypotheses on the phylogeny of the *Citrus* genus from other studies [2, 8]. Based on Fig 1, Mosambi and Qicheng had similar genetic profiles to sweet orange (*Citrus sinensis*), while Chanh Giay and Ma Nao Pan matched the genetic profile of Mexican lime (*Citrus aurantifolia*). Dalandan was located close to the other mandarins (*Citrus reticulata*), while Pontianak and Nagpur had an intermediate position between the mandarins and pomelos (*Citrus grandis)*. As a result, Chanh Giay and Ma Nao Pan were likely to be locally grown key limes (*Citrus aurantifolia*). Qicheng was classified as a sweet orange and Pontianak was likely a hybrid associated with mandarin, matching its species name (*Citrus nobilis*) which covers varieties that are hybrids of mandarin and oranges, otherwise known as tangors [7]. Notably, Dalandan and Nagpur also had strong associations to mandarins, despite being called oranges locally. Barkley et al. 2006 [23] previously reported Nagpur as a mandarin hybrid as well based on SSR markers. Despite having an appearance similar to limes, Mosambi was instead found to be genetically similar to Qicheng based on the analysis of the 16 SSR markers, suggesting that it is instead an orange hybrid.

**Fig 2:**
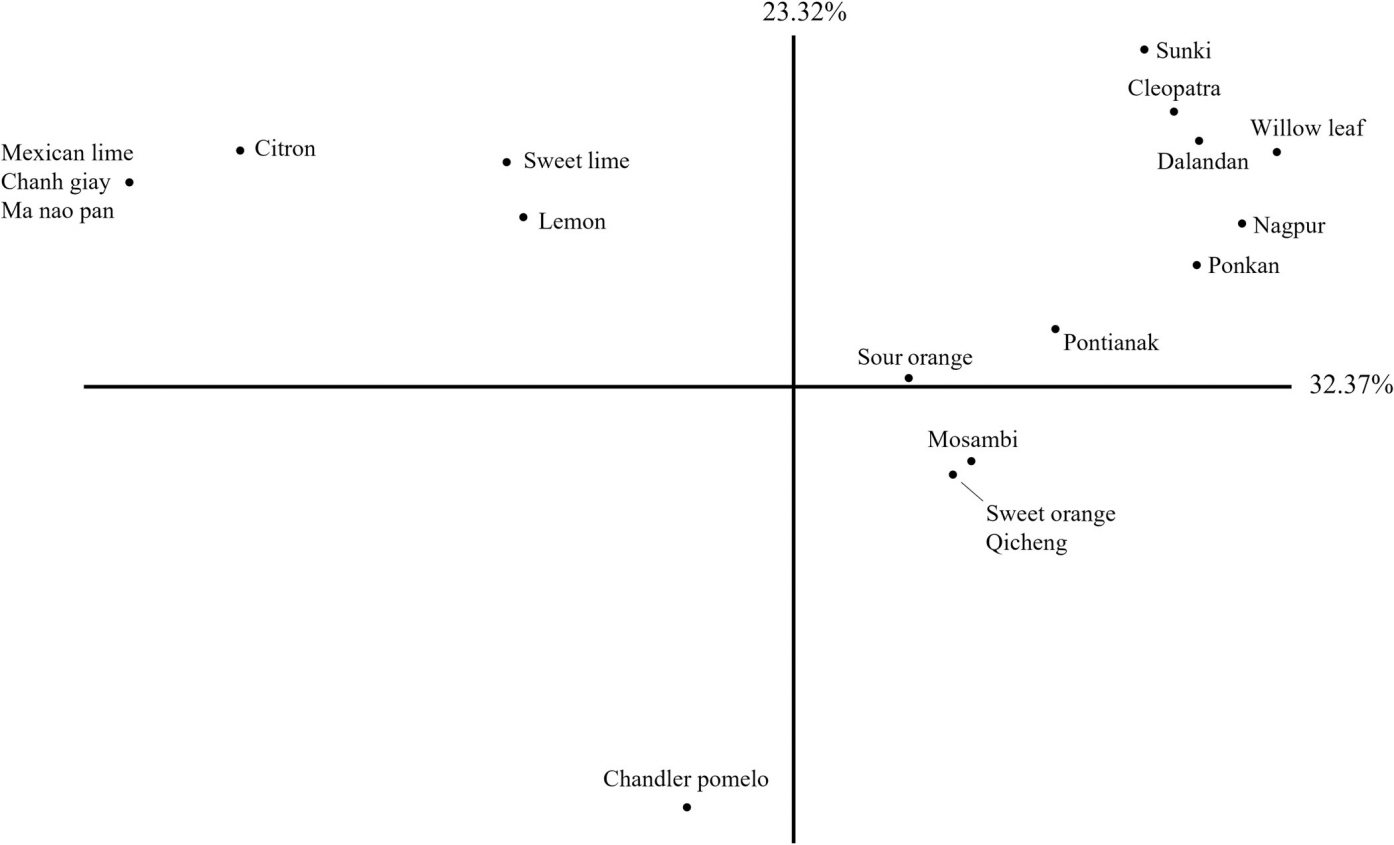
Factor analysis on genetic distance based on 16 SSR markers (S2(a) Table) of the seven Asian citrus varieties and their related accessions.

A factor analysis was then constructed for an in-depth visualisation of the determining SSR markers for the organisation of genetic variety (Fig 2). Clear separation was observed for various citrus varieties including the lime, lemon, orange and mandarin, indicating that the SSR markers were sufficient for the simple differentiation of the citrus varieties used. Like the NJ tree, Chanh Giay and Ma Nao Pan were clustered together. Dalandan and Nagpur were located in the top right quadrant with other known mandarin hybrids, while Pontianak could be considered a cluster with either the mandarins, or with sweet orange and sour orange. These match the NJ tree where these three species were closely related to the other known mandarins. Despite their shared mandarin origins, the extent of hybridisation would affect their similarities to each parental species–for example, Dalandan is more similar to a true mandarin even though both Dalandan and Pontianak had mandarin origins, likely due to different levels of backcrossing [24]. Pontianak thus may carry more orange-like traits than Dalandan and Nagpur. Mosambi was clustered with Qicheng and sweet orange, indicating that it is more likely to be a variety of orange (*Citrus sinensis*) than of sweet lime (*Citrus limetta*) as the NJ tree suggests, despite differences in its appearance with Qicheng. Based on the NJ tree in Fig 2, the separation of the seven varieties based on their SSR markers can be clearly visualised against the reference citrus varieties. The clustering pattern based on secondary metabolite profiles described in the next section would then be compared with the clusters observed in the NJ tree.

### 3.2. Secondary metabolites in citrus peel and juice

Analytical methods, especially gas and liquid chromatographic methods, have been used extensively for characterising the chemical profile of citrus fruits, although recent studies have expanded its use to detect adulteration and show of authenticity in processed citrus products such as juice concentrates [25–27]. This can also be extended to comparisons of citrus fruits of different species, as each species would have its own unique chemical fingerprint. Zhang et al. 2019 [28] identified four volatile compounds whose presence/absence could determine if a citrus germplasm belonged to the mandarin, sweet orange, lemon or pomelo species without the need for genetic analyses. While these four volatile compounds were able to categorise the germplasms with 90–100% accuracy, they were insufficient to differentiate between different varieties of citrus; for example, the fingered citron (*Citrus medica*) and Persian lime (*Citrus latifolia*) were both categorised as lemons (*Citrus limon*). As such, a broader spectrum of compounds may have to be considered when it comes to the nuanced differences between citrus hybrids.

Besides the presence of marker compounds, the abundance of these compounds can also differ greatly between similar varieties, creating a unique chemical makeup and thus a unique taste and aroma for each type of citrus. The ratio of secondary metabolites not only gives each variety its unique flavour profile, it also can serve as an indicator of its origins. In this section, both the non-volatile and volatile secondary metabolites were investigated to comprehensively capture the compositional diversity of the citrus fruits.

#### 3.2.1. Polyphenols, limonoids, coumarins and furanocoumarins in citrus peel

In addition to major non-volatile compounds such as sugars and organic acids, polyphenols, limonoids and furanocoumarins also play a role in the taste profile of citrus. These compounds are secondary metabolites whose production is regulated by gene expression and thus can vary between different varieties. A variety of glycosylated flavonoids were identified in this study, with varying abundances (Table 1, corresponding chemical properties and identifiers are listed in S3 Table). For most flavonoid compounds analysed in this study, similar concentrations were obtained for Chanh Giay and Ma Nao Pan, with major compounds being hesperidin, isorhamnetin-3-rutinoside and vicenin-2. Notably, there were some flavonoids unique to these two peel extracts, including kaempferol-3-rutinoside and vicenin-1. Some other flavonoids were only present in the remaining five species in low or trace amounts, such as eriocitrin, isorhamnetin-3-glucoside, isorhamnetin-3-rutinoside and isorhoifolin. On the other hand, didymin was detected in all the species (3–30 μg/mL) except for Chanh Giay and Ma Nao Pan. While variation was larger among Mosambi, Qicheng, Nagpur, Pontianak and Dalandan, there were still some similarities observed, such as lower levels of rutin and kaempferol-3-neohesperidoside in Mosambi, Qicheng and Nagpur, lower amounts of vitexin in Mosambi and Qicheng, and higher amounts of narirutin in Mosambi, Qicheng and Dalandan.

**Table 1:** Flavonoids, limonoids and coumarin compounds in citrus peel identified and quantified by LC-QTOF/MS (concentrations expressed in μg/mL).

No.	Compound	Mosambi	Qicheng	Nagpur	Pontianak	Dalandan	Chanh Giay	Ma Nao Pan
1	7-Methoxycoumarin	-	0.233 ± 0.021	0.511 ± 0.020	0.864 ± 0.025	0.519 ± 0.050	139.107 ± 2.607	61.053 ± 0.325
2	Bergamotine	-	-	-	-	-	0.729 ± 0.018	1.278 ± 0.031
3	Bergapten	-	-	-	-	-	332.617 ± 2.109	325.7 ± 6.243
4	Chrysoeriol-7-glucoside	-	Trace	Trace	9.725 ± 0.461	3.745 ± 0.165	-	-
5	Citropten	-	-	-	-	-	184.803 ± 2.754	186.65 ± 2.302
6	Didymin	30.187 ± 1.065	9.774 ± 0.065	6.905 ± 0.241	3.350 ± 0.098	5.195 ± 0.175	Trace	Trace
7	Eriocitrin	15.700 ± 0.746	9.398 ± 0.013	7.526 ± 0.181	8.621 ± 0.161	17.088 ± 0.120	50.190 ± 2.235	50.207 ± 0.625
8	Hesperidin	390.180 ± 5.080	312.347 ± 7.112	360.253 ± 5.934	342.843 ± 8.637	252.463 ± 8.734	166.387 ± 2.020	205.49 ± 3.061
9	Homoeriodicytol-7-glucoside	54.950 ± 1.958	42.911 ± 0.617	95.081 ± 1.473	57.437 ± 2.372	45.347 ± 3.124	35.837 ± 1.905	60.397 ± 1.854
10	Hyperoside	-	0.201 ± 0.005	0.183 ± 0.002	0.126 ± 0.005	0.600 ± 0.006	-	-
11	Isoquercetin	-	Trace	-	0.309 ± 0.036	0.834 ± 0.057	1.887 ± 0.0800	Trace
12	Isorhamnetin	-	-	-	-	-	Trace	Trace
13	Isorhamnetin-3-glucoside	-	0.080 ± 0.005	0.148 ± 0.002	0.114 ± 0.002	0.349 ± 0.005	3.106 ± 0.116	3.284 ± 0.292
14	Isorhamnetin-3-neohesperidoside	0.915 ± 0.058	0.459 ± 0.017	0.415 ± 0.022	0.996 ± 0.032	0.637 ± 0.030	-	-
15	Isorhamnetin-3-rutinoside	1.289 ± 0.099	0.811 ± 0.013	0.528 ± 0.044	3.367 ± 0.057	3.112 ± 0.068	161.477 ± 1.918	210.327 ± 3.031
16	Isorhoifolin	1.659 ± 0.042	1.380 ± 0.015	1.845 ± 0.092	0.061 ± 0.002	2.094 ± 0.031	8.443 ± 0.064	12.793 ± 0.050
17	Isoscoparin	0.960 ± 0.040	0.257 ± 0.007	2.398 ± 0.058	8.462 ± 0.167	1.655 ± 0.139	0.597 ± 0.025	0.715 ± 0.034
18	Isovitexin	0.412 ± 0.031	0.622 ± 0.020	1.890 ± 0.095	8.054 ± 0.110	1.797 ± 0.010	10.790 ± 0.594	15.898 ± 0.995
19	Kaempferol-3-neohesperidoside	13.650 ± 0.343	17.401 ± 0.065	14.081 ± 0.268	36.200 ± 0.480	49.573 ± 4.262	33.157 ± 0.845	38.770 ± 1.498
20	Kaempferol-3-rutinoside	-	-	-	-	-	20.903 ± 0.899	25.633 ± 0.915
21	Limonin	185.337 ± 4.969	28.399 ± 1.080	79.464 ± 1.412	79.960 ± 4.944	96.883 ± 7.278	29.133 ± 1.271	21.280 ± 1.337
22	Luteolin-6-glucoside	2.495 ± 0.107	4.708 ± 0.274	1.373 ± 0.082	50.560 ± 1.279	28.177 ± 1.333	2.636 ± 0.152	3.637 ± 0.135
23	Mangiferin	6.965 ± 0.157	29.038 ± 1.696	48.446 ± 2.666	67.615 ± 1.795	44.341 ± 0.954	-	-
24	Narirutin	375.887 ± 23.677	291.903 ± 23.962	95.610 ± 10.304	72.720 ± 2.627	184.603 ± 15.461	29.947 ± 1.678	64.770 ± 2.062
25	Neohesperidin	8.351 ± 0.304	9.428 ± 0.277	5.812 ± 0.090	5.156 ± 0.223	26.783 ± 0.476	6.055 ± 0.351	11.422 ± 0.273
26	Nobiletin	493.867 ± 7.606	430.233 ± 10.744	1842.400 ± 27.379	4014.867 ± 40.611	4601.533 ± 48.065	0.055 ± 0.002	0.243 ± 0.004
27	Nomilin	61.953 ± 0.571	-	6.563 ± 0.712	6.304 ± 0.102	16.336 ± 0.504	79.348 ± 3.697	35.499 ± 2.209
28	Obacunone	-	-	2.518 ± 0.177	4.428 ± 0.187	3.051 ± 0.083	-	-
29	Orientin	0.922 ± 0.064	1.383 ± 0.004	4.865 ± 0.168	23.857 ± 0.577	15.364 ± 0.241	1.602 ± 0.100	3.032 ± 0.136
30	Prunin	-	0.930 ± 0.051	0.294 ± 0.017	0.098 ± 0.006	1.712 ± 0.052	-	-
31	Quercetin-3-neohesperidoside	-	0.082 ± 0.002	0.225 ± 0.015	1.418 ± 0.062	0.829 ± 0.020	-	-
32	Rhoifolin	-	-	-	-	Trace	-	-
33	Rutin	3.612 ± 0.101	4.106 ± 0.128	6.063 ± 0.127	26.043 ± 0.749	26.523 ± 1.414	35.443 ± 0.449	29.630 ± 0.770
34	Scoparone	1.098 ± 0.078	17.819 ± 0.142	2.292 ± 0.093	0.639 ± 0.007	0.214 ± 0.011	1.412 ± 0.049	1.720 ± 0.049
35	Sinensetin	167.667 ± 3.313	270.500 ± 5.469	172.133 ± 1.790	94.317 ± 1.582	31.867 ± 0.147	Trace	Trace
36	Tangeretin	44.163 ± 0.522	56.100 ± 0.361	594.433 ± 1.464	667.067 ± 2.495	1374.067 ± 12.055	Trace	0.058 ± 0.001
37	Vicenin-1	-	-	-	-	-	112.827 ± 0.766	172.483 ± 0.884
38	Vicenin-2	273.910 ± 6.057	261.980 ± 8.861	760.967 ± 14.364	1956.967 ± 53.806	736.700 ± 60.789	179.517 ± 1.814	253.417 ± 4.003
39	Vitexin	0.860 ± 0.031	0.638 ± 0.080	3.151 ± 0.231	11.054 ± 0.884	3.146 ± 0.103	11.960 ± 0.850	22.783 ± 1.801

Other than glycosylated flavonoids, another subclass of flavonoids, polymethoxyflavones (PMF) also accounted for a large portion of all the peels except for Chanh Giay and Ma Nao Pan peels, where they were detected at trace levels. A few trends can be noted from the PMF compounds present in the other five peels: Dalandan contained the lowest amount of sinensetin (approximately 32 μg/mL); Mosambi and Qicheng had relatively similar nobiletin concentrations (~494 and 430 μg/mL, respectively) while Pontianak and Dalandan were almost 10 times higher in nobiletin; Mosambi and Qicheng had the lowest tangeretin concentration (~44 and 56 μg/mL, respectively), followed by Nagpur and Pontianak (~594 and 667 μg/mL) and finally Dalandan which had approximately 1374 μg/mL of tangeretin. Among the three mandarin hybrids, Dalandan could be identified based on its lower sinensetin and higher tangeretin concentrations, while Nagpur had a nobiletin concentration intermediate of the other mandarin hybrids and Mosambi/Qicheng. Pontianak had a combination of the above trends–it had a similar nobiletin concentration to Dalandan, while having a similar tangeretin concentration to Nagpur, all of which were present in Mosambi and Qicheng in lower concentration.

While the amount of PMF compounds was insignificant in Chanh Giay and Ma Nao Pan peels, they were found to contain several coumarin and furanocoumarin compounds which were largely absent in the other peels. 7-Methoxycoumarin was detected in Chanh Giay and Ma Nao Pan peels at concentrations of about 139 and 61 μg/mL, respectively, while they were present in amounts less than 1 μg/mL in the other peels. Notably, Qicheng contained the highest amount of scoparone. While high concentrations of bergapten and citropten were detected in Chanh Giay and Ma Nao Pan peels, they were absent in the others; similarly, bergamotine was only detected in these two peels, although at much lower concentrations. Of the limonoids analysed, obacunone was only found in the peel of the three mandarin hybrids (Nagpur, Pontianak, Dalandan). Limonin was found in all peels; however, Mosambi had a significantly higher amount of limonin compared to the others.

Based on the distribution of polyphenols, limonoids, coumarins and furanocoumarins, PMF compounds and furanocoumarins are standout markers for differentiation between the different species of citrus. PMF compounds such as sinensetin and nobiletin clearly distinguished Chanh Giay and Ma Nao Pan from the other five varieties, similarly furanocoumarins like bergapten and citropten were only present in these two varieties. Similarities between Chanh Giay and Ma Nao Pan were expected as they are both regarded locally as common limes and have been shown by their SSR markers to potentially be of the same species. Generally, Nagpur, Pontianak and Dalandan shared many trends in their secondary metabolite profile; however, there were some clear differences in their concentrations of PMF compounds which could be due to different parental varieties. Lastly, similarities between Mosambi and Qicheng agreed with conclusions from their SSR markers that the two were more closely related to the orange than to lime or mandarin, although there were still differences in their secondary metabolite profile for some compounds such as limonin.

#### 3.2.2. Polyphenols, limonoids, coumarins, furanocoumarins and xanthones in citrus juice

The composition of the juice of the seven citrus varieties is shown in Table 2 (corresponding chemical properties and identifiers are listed in S3 Table). An important class of non-volatiles in citrus juices is limonoids, as the limonoid aglycones are known to impart bitter taste to the juice. Limonin and nomilin are the two most abundant limonoids and are not only often used as indicators of bitterness, but also as a marker for maturation [29, 30]. Chanh Giay and Ma Nao Pan were found to have approximately three times higher limonin than the other five species, while Mosambi and Nagpur had the highest nomilin content. This could contribute to the bitterness of the juices of these varieties. Like the peel, furanocoumarins were abundant in Chanh Giay and Ma Nao Pan, namely bergamotine and bergapten, which were absent in all other species studied. A similar trend was also observed for PMFs–Chanh Giay and Ma Nao Pan generally contained lower amounts of these compounds, especially for sinensetin and nobiletin. Notably, Mosambi and Qicheng were observed to contain relatively similar concentrations (sinensetin: ~0.086 μg/mL and nobiletin: ~0.183 μg/mL in both Mosambi and Qicheng).

**Table 2:** Flavonoids, limonoids and coumarin compounds in citrus juice identified and quantified by LC-QTOF/MS (concentrations expressed in μg/mL).

No.	Compound	Mosambi	Qicheng	Nagpur	Pontianak	Dalandan	Chanh Giay	Ma Nao Pan
1	Bergamotine	-	-	-	-	-	11.194 ± 0.270	9.204 ± 0.093
2	Bergapten	-	-	-	-	-	5.896 ± 0.119	6.068 ± 0.054
3	Chrysoeriol-7-glucoside	-	-	-	-	-	Trace	Trace
4	Citropten	-	-	-	-	-	2.610 ± 0.058	6.856 ± 0.058
5	Didymin	6.805 ± 0.182	7.087 ± 0.155	6.678 ± 0.085	3.379 ± 0.134	5.385 ± 0.218	0.209 ± 0.006	0.165 ± 0.018
6	Eriocitrin	1.138 ± 0.038	0.919 ± 0.031	0.566 ± 0.019	0.875 ± 0.032	0.652 ± 0.047	9.238 ± 0.148	3.848 ± 0.050
7	Hesperidin	54.883 ± 1.470	50.796 ± 1.731	43.654 ± 0.456	58.066 ± 0.510	55.331 ± 1.194	39.940 ± 1.518	31.193 ± 1.042
8	Homoeriodicytol-7-glucoside	6.657 ± 0.274	5.844 ± 0.086	6.372 ± 0.246	6.196 ± 0.133	5.851 ± 0.283	5.299 ± 0.112	5.242 ± 0.081
9	Isomangiferin	-	-	-	-	-	0.230 ± 0.020	0.119 ± 0.012
10	Isoquercetin	-	-	-	-	-	Trace	0.275 ± 0.008
11	Isorhamnetin	-	-	-	-	-	Trace	Trace
12	Isorhamnetin-3-glucoside	Trace	-	-	-	-	0.071 ± 0.002	0.095 ± 0.000
13	Isorhamnetin-3-neohesperidoside	-	-	-	-	-	0.066 ± 0.003	0.047 ± 0.002
14	Isorhamnetin-3-rutinoside	0.431 ± 0.024	0.306 ± 0.027	Trace	0.149 ± 0.012	Trace	2.582 ± 0.237	5.432 ± 0.119
15	Isorhoifolin	0.042 ± 0.001	0.047 ± 0.005	Trace	Trace	0.056 ± 0.003	0.513 ± 0.012	0.124 ± 0.002
16	Kaempferol-3-neohesperidoside	0.083 ± 0.002	0.055 ± 0.001	0.092 ± 0.004	0.022 ± 0.000	0.052 ± 0.005	1.149 ± 0.035	0.822 ± 0.016
17	Limonin	6.313 ± 0.090	1.296 ± 0.033	5.405 ± 0.080	5.530 ± 0.165	3.815 ± 0.082	16.020 ± 0.518	15.088 ± 0.322
18	Luteolin-6-glucoside	Trace	0.122 ± 0.002	-	Trace	Trace	0.080 ± 0.003	0.071 ± 0.004
19	Mangiferin	-	2.250 ± 0.140	3.180 ± 0.128	3.429 ± 0.147	1.696 ± 0.011	-	-
20	Narirutin	20.339 ± 0.453	43.655 ± 2.028	17.112 ± 0.438	22.456 ± 0.117	27.358 ± 1.024	1.360 ± 0.046	1.057 ± 0.078
21	Neohesperidin	0.238 ± 0.013	0.267 ± 0.011	0.228 ± 0.016	0.140 ± 0.006	0.645 ± 0.006	1.518 ± 0.024	1.209 ± 0.019
22	Nobiletin	0.183 ± 0.004	0.183 ± 0.002	0.311 ± 0.010	0.350 ± 0.008	0.105 ± 0.002	0.006 ± 0.000	0.035 ± 0.001
23	Nomilin	3.967 ± 0.029	-	2.847 ± 0.053	2.117 ± 0.058	1.104 ± 0.035	1.578 ± 0.017	1.980 ± 0.049
24	Prunin	-	0.066 ± 0.002	-	-	0.203 ± 0.007	-	-
25	Quercetin-3-neohesperidoside	-	-	-	-	-	0.108 ± 0.003	0.103 ± 0.004
26	Rutin	1.017 ± 0.018	0.541 ± 0.011	0.108 ± 0.003	0.260 ± 0.003	0.148 ± 0.003	1.576 ± 0.014	2.544 ± 0.026
27	Scoparone	Trace	-	Trace	-	-	Trace	0.051 ± 0.000
28	Sinensetin	0.086 ± 0.001	0.086 ± 0.001	0.025 ± 0.001	0.014 ± 0.000	-	0.010 ± 0.000	0.013 ± 0.000
29	Tangeretin	0.031 ± 0.001	0.035 ± 0.000	0.051 ± 0.001	0.155 ± 0.005	0.031 ± 0.001	0.012 ± 0.000	0.031 ± 0.000
30	Vicenin-1	-	-	-	-	-	0.159 ± 0.007	0.307 ± 0.007
31	Vicenin-2	19.777 ± 0.680	12.545 ± 0.501	14.331 ± 0.314	25.644 ± 0.238	1.322 ± 0.050	5.018 ± 0.046	3.036 ± 0.219
32	Vitexin	Trace	-	-	-	-	0.113 ± 0.009	0.112 ± 0.007

For glycosylated flavonoids, some trends were observed in the juice that were not present in the peel. For example, while Dalandan had significantly higher amounts of neohesperidin than the other six in the peel, Chanh Giay and Ma Nao Pan had the highest neohesperidin content in the juice. Pontianak and Dalandan had comparable amounts of rutin to that of Ma Nao Pan (26–29 μg/mL) in the peel, however they had much lower levels of rutin in the juice. Narirutin was the highest in Mosambi peel, but Qicheng had the highest narirutin content in the juice (~44 μg/mL). Other trends however remained the same, such as eriocitrin and isorhamnetin-3-rutinoside. Generally, significant differences in the composition can be observed between the limes (Chanh Giay and Ma Nao Pan) and the orange/mandarin hybrids. Like the peel and SSR markers, Mosambi was found to have a more similar profile with the orange/mandarin hybrids than the lime, showing that it could be from an accession closer to the orange or mandarin families. From the trends observed in Table 2, several flavonoids such as neohesperidin and didymin can be useful for distinguishing different varieties of citrus alongside the PMF compounds and furanocoumarins.

#### 3.2.3. Key volatile compounds in citrus peel

Terpenes are the major compound class in all citrus volatile profiles; although while few terpene hydrocarbons contribute significantly to the aroma, many of their oxygenated derivatives are key odourants [31, 32]. The concentration of some key citrus volatiles reported in lime, mandarin, and orange as well as the composition sorted by each volatile class are shown in Table 3 (classification are divided into terpene derivatives and non-terpene compounds guided by Tisserand & Young 2014 [33]; key citrus volatiles are selected from previous studies of citrus volatiles [6, 34–37]).

**Table 3:** Key volatile compounds (expressed in μg/mL) and volatile compounds sorted by functional class (expressed in percentage of total concentration (%)) in citrus peel.

No.	Compound	Mosambi	Qicheng	Nagpur	Pontianak	Dalandan	Chanh Giay	Ma Nao Pan
1	α-Pinene	8.558 ± 0.881	36.121 ± 2.808	61.431 ± 3.456	52.674 ± 0.762	166.66 ± 24.511	8.583 ± 2.068	3.179 ± 0.335
2	Limonene	3535.873 ± 430.506	7250.732 ± 340.933	8189.164 ± 308.533	9332.113 ± 830.904	8285.298 ± 774.947	725.620 ± 150.105	372.073 ± 29.305
3	Terpinolene	2.923 ± 0.232	6.534 ± 0.661	16.064 ± 1.326	3.234 ± 0.239	42.295 ± 3.097	3.046 ± 0.253	0.320 ± 0.005
4	Citronellal	1.786 ± 0.108	2.997 ± 0.102	1.778 ± 0.135	1.854 ± 0.177	6.367 ± 0.273	-	0.198 ± 0.012
5	Linalool	6.772 ± 0.250	20.702 ± 1.352	14.932 ± 1.265	20.024 ± 1.525	22.892 ± 1.524	6.874 ± 0.567	3.254 ± 0.135
*6*	*trans*-α-Bergamotene	-	-	-	-	-	24.368 ± 1.441	3.509 ± 0.111
7	β-Caryophyllene	0.360 ± 0.140	1.883 ± 0.095	0.128 ± 0.015	0.510 ± 0.014	7.680 ± 0.220	35.746 ± 1.809	3.906 ± 0.481
8	Terpinen-4-ol	0.224 ± 0.032	0.423 ± 0.021	2.428 ± 0.225	1.340 ± 0.155	19.301 ± 1.048	-	-
9	Neral	1.604 ± 0.076	4.224 ± 0.123	-	0.807 ± 0.070	0.613 ± 0.024	11.678 ± 0.933	3.516 ± 0.136
10	α-Farnesene	-	-	0.615 ± 0.055	-	23.555 ± 2.646	32.234 ± 3.199	15.705 ± 2.032
11	Geranial	2.513 ± 0.205	6.828 ± 0.321	-	1.395 ± 0.083	-	-	4.377 ± 0.272
12	Citronellol	2.276 ± 0.251	3.141 ± 0.191	2.706 ± 0.335	2.360 ± 0.329	8.137 ± 0.551	-	0.510 ± 0.085
13	Nerol	1.325 ± 0.178	4.638 ± 0.209	0.595 ± 0.063	6.485 ± 0.190	5.588 ± 0.352	7.093 ± 0.746	3.350 ± 0.354
14	Perillyl aldehyde	0.772 ± 0.046	0.666 ± 0.032	2.873 ± 0.181	2.611 ± 0.194	1.646 ± 0.215	0.738 ± 0.208	0.341 ± 0.040
15	Geraniol	0.952 ± 0.058	4.174 ± 0.350	-	0.732 ± 0.067	0.989 ± 0.036	8.914 ± 0.925	5.235 ± 0.266
16	Nerolidol	0.053 ± 0.014	0.170 ± 0.006	0.165 ± 0.008	0.038 ± 0.007	0.263 ± 0.041	0.254 ± 0.030	0.310 ± 0.013
17	α-Sinensal	0.245 ± 0.026	2.021 ± 0.119	1.299 ± 0.125	0.044 ± 0.005	69.937 ± 5.208	-	0.002 ± 0.000
*1*	*Total terpene hydrocarbons*	99.285	98.553	98.976	99.039	97.546	93.899	90.656
*2*	*Total terpene derived acids*	0.005	0.003	<0.001	0.001	0.004	0.104	0.083
*3*	*Total terpene derived alcohols*	0.419	0.618	0.429	0.547	0.986	4.076	5.537
*4*	*Total terpene derived aldehydes*	0.178	0.261	0.071	0.069	0.789	0.746	1.283
*5*	*Total terpene derived esters*	0.022	0.041	-	0.028	0.068	-	-
*6*	*Other oxygenated terpenes*	0.031	0.050	0.101	0.030	0.069	0.506	0.895
*7*	*Total acids*	0.017	0.067	0.023	0.014	0.026	0.069	0.133
*8*	*Total alcohols*	0.020	0.131	0.105	0.080	0.288	0.140	0.480
*9*	*Total aldehydes*	0.021	0.256	0.290	0.190	0.212	0.443	0.799
*10*	*Total esters*	<0.001	0.006	<0.001	<0.001	0.001	-	-
*11*	*Others*	0.002	0.013	0.003	0.002	0.010	0.017	0.134

In line with previous studies, limonene was the major compound detected in our study, accounting for approximately 94–97% of volatile compounds in Mosambi, Qicheng, Nagpur and Pontianak, 86% in Dalandan and 51% in Chanh Giay and Ma Nao Pan [32]. The lower levels of limonene in the two lime varieties match that of the volatiles reported in lime where they can contain lower percentages of limonene, averaging 61% across 21 lime species in a study by Lota, Serra, Tomi, Jacquemond & Casanova 2002 [38]. Comparatively, oranges, mandarins and their hybrids have higher amounts of limonene, especially sweet oranges which were reported to have a limonene content range of 88–95% [32, 39].

Other than limonene, notable terpenes included *trans-*α-bergamotene, which was found in significant amounts in Chanh Giay (approximately 24 μg/mL) and Ma Nao Pan (~4 μg/mL) but absent in the other species. This matches reports in literature where *trans*-α-bergamotene is more abundant in key limes (*Citrus aurantifolia*) than other citrus species, concurring with the SSR analysis where these two varieties were suggested to be key limes [6]. Despite having much lower amounts of limonene, with only approximately 372 μg/mL detected in Ma Nao Pan compared to 9332 μg/mL in Pontianak (highest), the overall percentage of total terpenes remains high in all seven varieties, the lowest being 93.9% and 90.7% in Chanh Giay and Ma Nao Pan, respectively.

Oxygenated terpene derivatives such as terpene alcohols and aldehydes are the next largest class, with significantly higher percentages in Chanh Giay and Ma Nao Pan. Neral was found to be abundant in Chanh Giay (~12 μg/mL), while geranial was most abundant in Qicheng (~7 μg/mL), and absent in Chanh Giay, Dalandan and Nagpur. Nerol was highest in Pontianak and Chanh Giay (6–7 μg/mL), and geraniol was highest in Chanh Giay and Ma Nao Pan (5–9 μg/mL). While these compounds were dominant in Chanh Giay and Ma Nao Pan, citronellal and citronellol were largely absent or in trace amounts. Compared to the other six varieties, Dalandan had the highest amount of citronellal, (~6 μg/mL), citronellol (~8 μg/mL), terpinen-4-ol (~19 μg/mL) and α-sinensal (~70 μg/mL). α-Sinensal is of note as it is a key indicator for the differentiation between mandarins (≥ 0.7%) and oranges (< 0.05%), suggesting that Dalandan is closer to a mandarin than the other non-lime varieties [6]. Based on the terpenes and their derivatives, Dalandan stood out as with a unique profile, while Chanh Giay and Ma Nao Pan were similar. Mosambi, Qicheng, Nagpur and Pontianak had lesser notable differences between their volatile profiles, although the high limonene content would point them towards being related to the orange or mandarin. Overall, high abundance terpenes such as limonene and total terpene hydrocarbons were useful in distinguishing limes from mandarin or orange varieties, while citronellal, citronellol and α-sinensal could be employed for the differentiation of lime, mandarin and orange varieties.

### 3.3. Correlation between genetic and secondary metabolites composition in citrus

**Fig 3:**
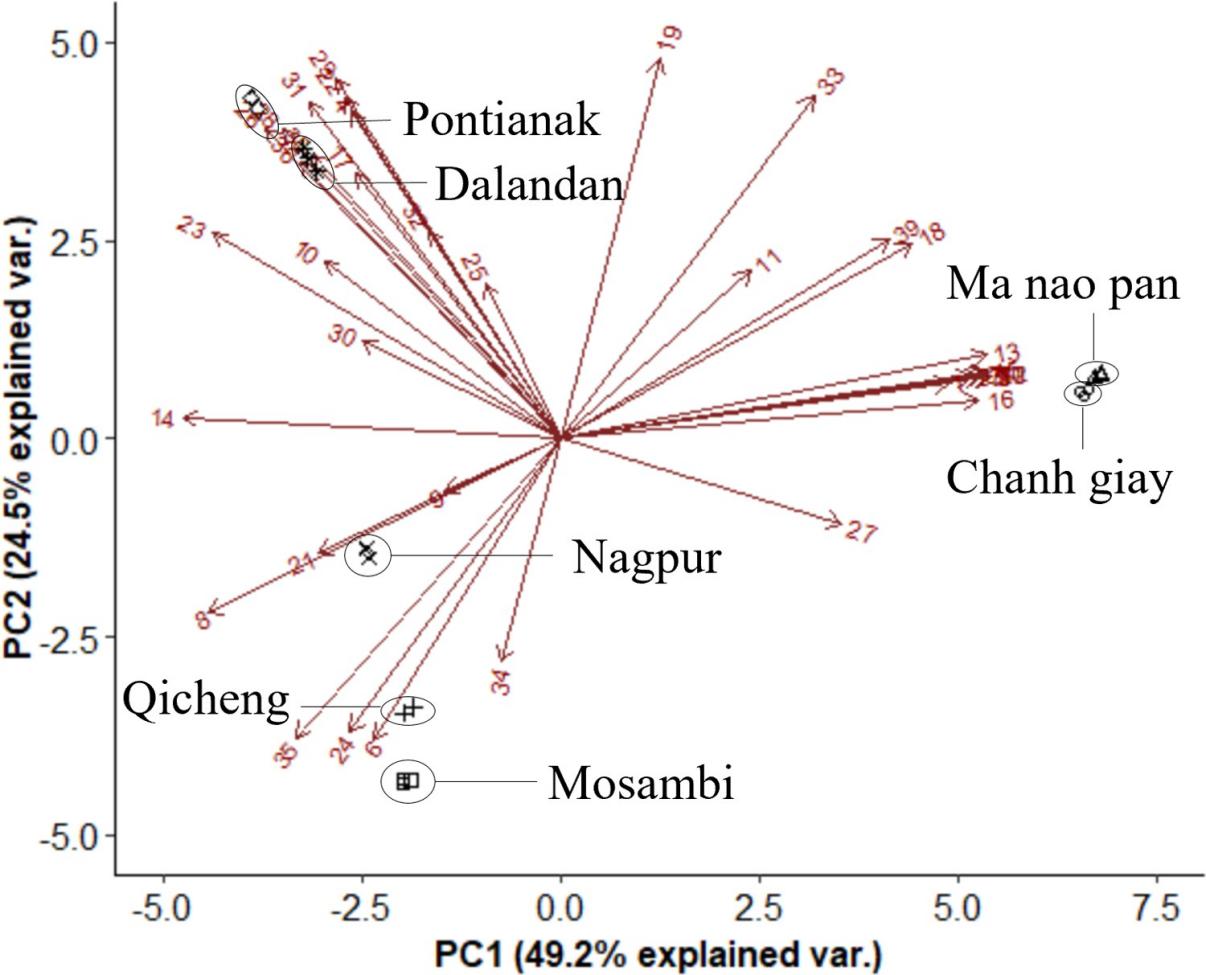
Biplots of identified non-volatile secondary metabolites in peel extracts of seven Asian citrus varieties. Numbers on the biplots correspond to Table 1.

**Fig 4:**
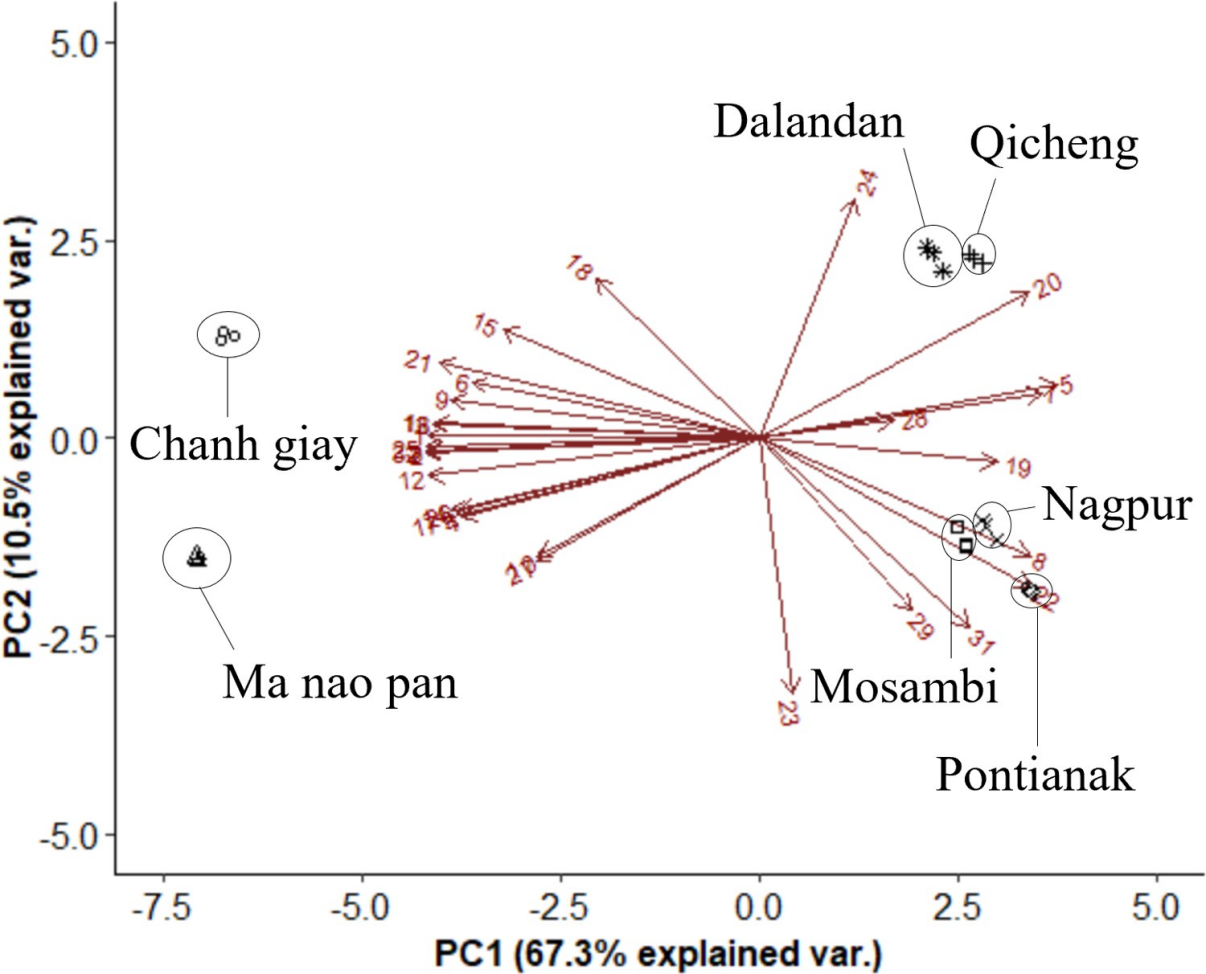
Biplots of identified non-volatile secondary metabolites in juice extracts of seven Asian citrus varieties. Numbers on the biplots correspond to Table 2.

While analyses of the genetic markers may provide some insights into their accessions, it does not fully reflect differences in their flavour profile which is dependent on their chemical composition. Figs 3 and 4 show the clustering of the seven different varieties in their peel and juice, respectively, based on their secondary metabolite profile. The non-volatile composition of the peels of the seven investigated citrus varieties appeared to correlate well with their genetic backgrounds. From Fig 3, the lime species and the orange/mandarin hybrids were clearly separated along PC1, which accounted for 49.7% of the variation. Many compounds contributed to separation in the PC1 axis, such as bergapten, isorhamnetin glucosides and vicenin-1, where significant differences in abundance between the limes and other species were observed for these compounds. Pontianak and Dalandan were separated from Nagpur, Qicheng and Mosambi along PC2, due to compounds such as limonin, sinensetin and vicenin-2. Although the variation supported by PC2 was only 24.3%, visual differentiation was still observed between oranges and mandarins along this PC. Qicheng and Mosambi were found to be located close together in the negative PC1 and PC2 quadrant, matching their genetic profiles.

The limes and orange/mandarin hybrids were similarly separated significantly along PC1 (67.3% of the variation explained in PC1) in Fig 4, which charted the non-volatile components (flavonoids, limonoids, coumarins) in the juice. The compounds responsible for the separation were mainly coumarins as well as some flavonoids and limonoids including didymin, isorhamnetin glucosides and limonin. Slight separation was observed along PC2, although the low explained variation percentage (10.5%) suggested that the two major clusters were still between the limes and orange/mandarin hybrids. More similarity was observed between the juice profiles of Dalandan and Qicheng and between Nagpur, Mosambi and Pontianak, which was different from the non-volatile profile of the peel. As secondary metabolites in the peel and juice are synthesised independently, different peel and juice profiles for each citrus can be expected.

**Fig 5:**
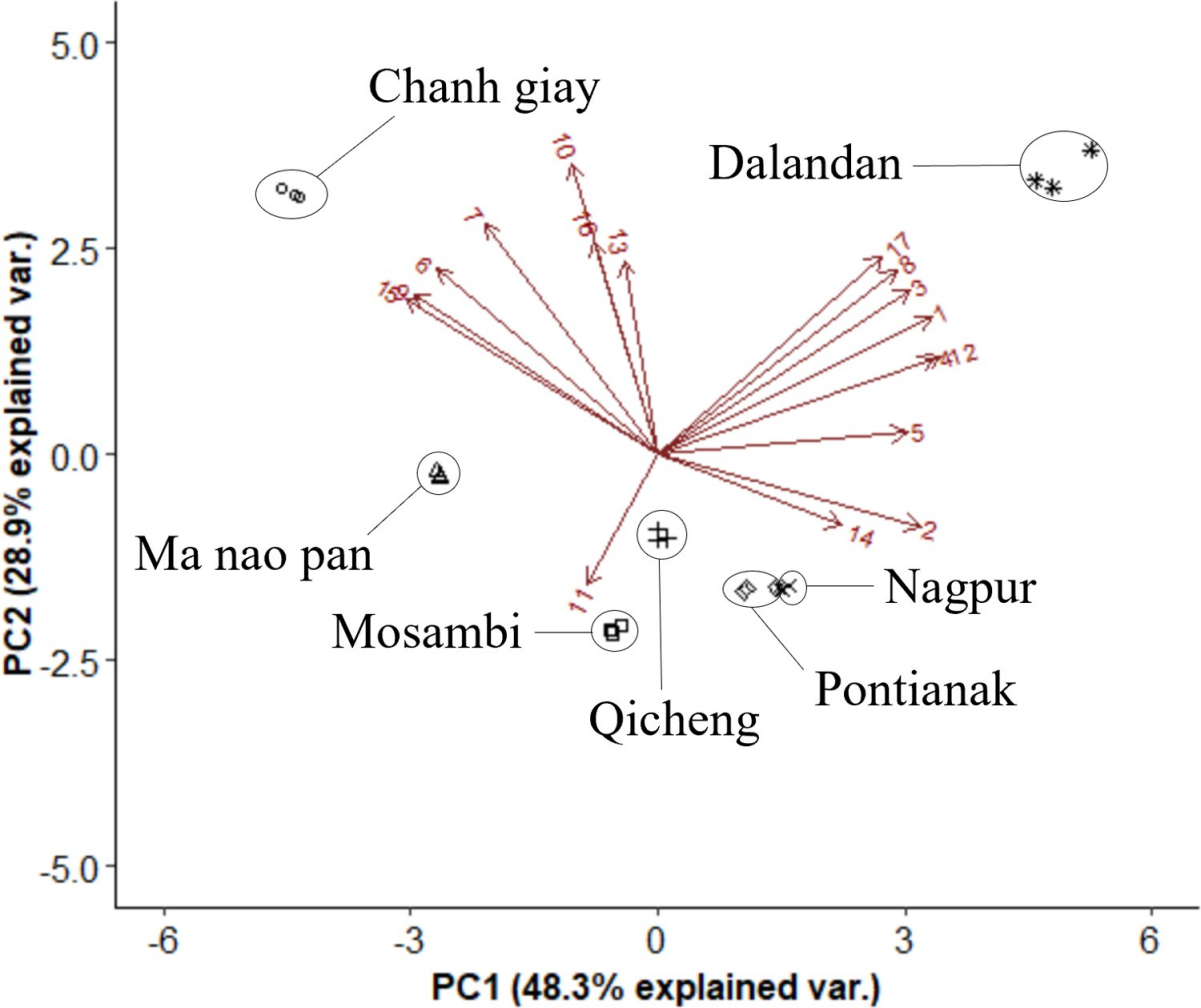
Biplot of key volatile compounds in the peel of seven Asian citrus varieties. Numbers on the biplots correspond to Table 3.

**Fig 6:**
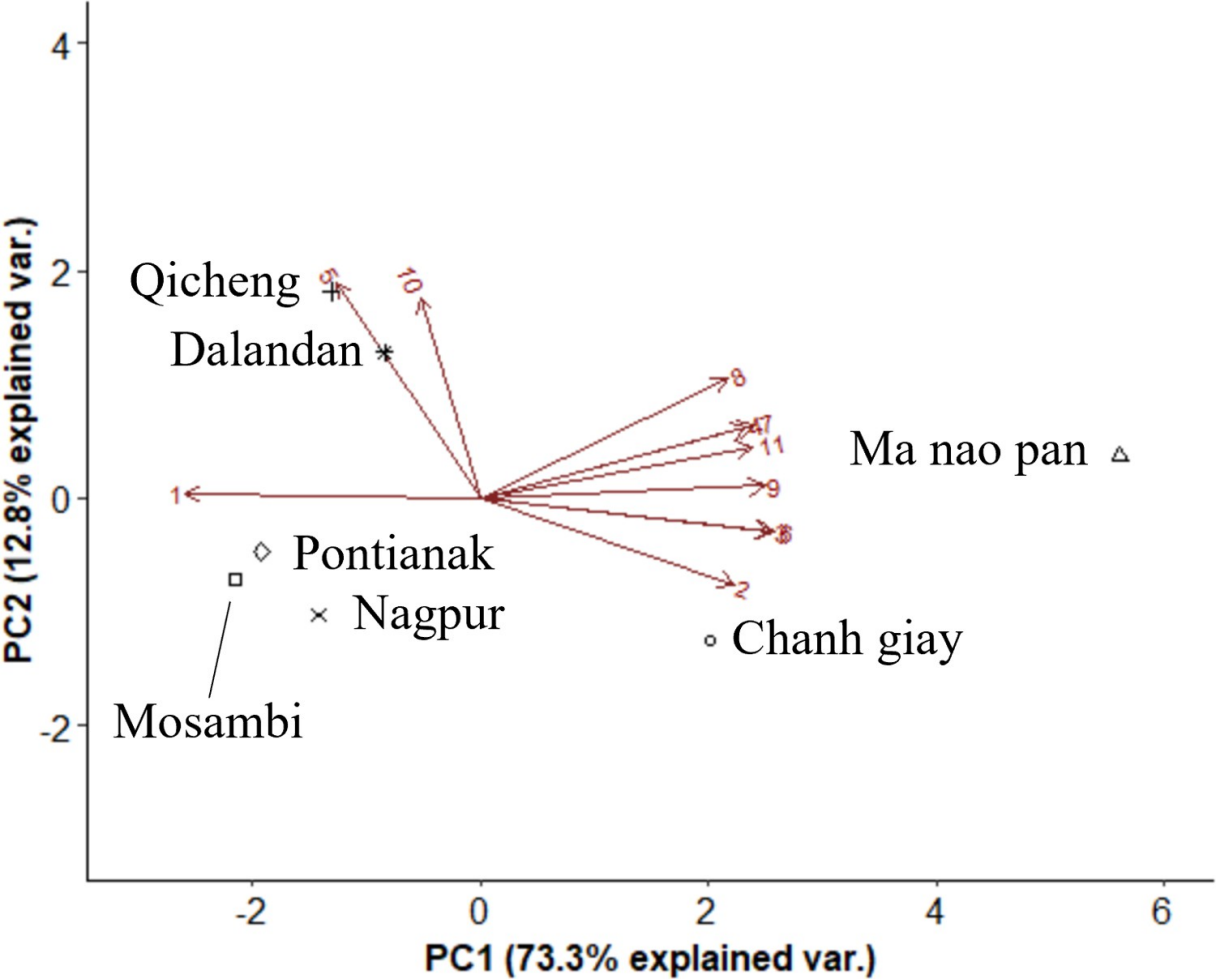
Biplot of volatile compounds in the peel of seven Asian citrus varieties sorted by functional class. Numbers on the biplots correspond to Table 3.

Figs 5 and 6 further demonstrate the clustering using the volatile profiles of the seven citrus varieties. A total of 18 volatile compounds commonly associated with citrus were selected and plotted in Fig 5, which showed three clusters: the limes, Dalandan and the remaining species, with 48.3% of the variation explained in PC1. Further separation was seen between Chanh Giay and Ma Nao Pan, and between Dalandan and the remaining four varieties, along PC2 (28.9%). The differences in concentrations of key odourants like α-pinene, terpinolene, citronellal, terpinen-4-ol, citronellol and α-sinensal in Dalandan contributed significantly to the separation of Dalandan from the other non-lime varieties, suggesting that the aroma profile of Dalandan may be perceived differently from the other varieties. Despite having similar non-volatile profiles, some separation was observed between Chanh Giay and Ma Nao Pan along PC2, due to compounds such as β-caryophyllene, α-farnesene and nerolidol. This does not match the clustering observed from the genetic profile, where Dalandan was grouped closely with Nagpur, and Chanh Giay with Ma Nao Pan. The variation in abundances of these volatiles thus resulted in different clustering patterns compared to their genetic profiles.

Fig 6 shows a PCA biplot of the seven citrus varieties differentiated by the composition of different volatile compound groups. Three clusters were observed to be separated by PC1 (73.3%). Notably, Chanh Giay and Ma Nao Pan were separated significantly along PC1, due to the differences in their percentage of oxygenated terpene derivatives of the acids, alcohol and oxide classes as well as non-terpene aldehydes. The remaining five varieties had slight separation along PC2 which only explained 12.8% of the variation, mainly due to the terpene esters and simple esters in Qicheng and Dalandan. Like Fig 3, the clustering for Chanh Giay and Ma Nao Pan differed greatly from that in the Fig 2. The different volatile composition in the two limes could be attributed to factors such as agricultural or environmental differences, given that they were grown in two different countries. While separation between the oranges and mandarin hybrids was observed in Fig 2, such differentiation was not clear based on volatile profiles, except for Dalandan.

These results show that variations in secondary metabolite profiles could be present even in citrus fruits that are genetically closely related, which could be due to different environment factors or agricultural practices in their respective countries. Additionally, mutations arising in these varieties that affect their metabolic pathway and therefore production of secondary metabolites would not be reflected in their SSR marker profile. Notably, secondary metabolites in the peels were observed to display similar clustering to the genetic profiles, and this correlation can be explored in the future for a creation of a model to understand different citrus varieties based on their non-volatile composition.

## 4. Conclusion

Genetic and compositional analyses of seven citrus varieties grown in Asia showed that genetically similar varieties can have very different volatile or non-volatile profiles, which can greatly affect the perceived flavour of these hybrids by consumers. The use of both genetic and compositional analyses in parallel to study citrus varieties was suggested to obtain a greater understanding of their origins and secondary metabolite profiles. Of the seven varieties studied, Chanh Giay and Ma Nao Pan were found to have similar genetic (Mexican lime) and non-volatile profiles, but significantly different volatile profiles. Three mandarin hybrids studied, Dalandan, Nagpur and Pontianak, also had different volatile and non-volatile profiles despite all having genetic profiles suggesting they were derived from mandarins. Notably, Mosambi and Qicheng had similar non-volatile and volatile profiles and were both genetically sweet oranges. The importance of genetic studies for the tracing of citrus accessions and discovery of the origins of different citrus varieties was revealed by observing that the Mosambi used in this study was of the *Citrus sinensis* (orange) species, instead of *Citrus limetta* (sweet lime/lemon) based on genetic data. However, studies on the secondary metabolite profile are equally important to provide knowledge of the taste and aroma profiles of these varieties as these profiles can be significantly different even in genetically similar varieties, such as in the two Mexican limes studied. Non-volatile compounds such as polymethoxyflavones, furanocoumarins and volatile compounds such as citronellol and α-sinensal were some of the major secondary metabolites that contributed to major differences in the secondary metabolite profiles, and these compounds could be further studied as markers for differentiation based on species or environmental conditions. Using both genetic and compositional means to study complex citrus varieties would gain further insights into both the origins and flavour constituents of these varieties, which could then be extended to screen for unique flavour compounds for applications in citrus flavour development.

## Supporting information

S1 Table Citrus varieties used as references for the genetic origin analysis. (DOCX)

S2 Table (a) Descriptions of the 16 SSR markers used. (b) Allelic sizes of the amplified DNA fragments at the 16 loci of SSR for the studied citrus genotypes.
(ZIP)

S3 Table Chemical properties of compounds in Tables 1 and 2 used for identification. (DOCX)
