# Supplementary material for: Distinguishing citrus varieties based on genetic and compositional analyses
Source: PLoS One. 2022 Apr 18;17(4):e0267007. doi: 10.1371/journal.pone.0267007 (PMC9015143; doi:10.1371/journal.pone.0267007)
Supplement: S1 Table — (DOCX) [file pone.0267007.s001.docx]

**Distinguishing citrus varieties based on genetic and compositional analyses**

**Rui Min Vivian Goh^a^, Aileen Pua^a,b^, Francois Luro^c^, Kim Huey Ee^b^, Yunle Huang^a,b^, Elodie Marchi^c^, Shao Quan Liu^a*^, Benjamin Lassabliere^b^, Bin Yu^b^^[[1]](#footnote-1)^***

^a^Department of Food Science and Technology, National University of Singapore, S14 Level 5, Science Drive 2, Singapore 117542

^b^Mane SEA PTE LTD, 3 Biopolis Drive, #07-17/18/19 Synapse, Singapore 138623

^c^UMR AGAP Institut, Univ Montpellier, CIRAD, INRAE, Institut Agro -, 20230, San Giuliano, France

Supplementary Table S1. Citrus varieties used as references for the genetic origin analysis

| **Citrus name** | **Swingle classification** | **Origin** |
| --- | --- | --- |
| Sunki (mandarin) | *C. reticulata* Blanco | INRAE-CIRAD BRC |
| Ponkan (mandarin) | *C. reticulata* Blanco | INRAE-CIRAD BRC |
| Cleopatra (mandarin) | *C. reticulata* Blanco | INRAE-CIRAD BRC |
| Willow leaf (mandarin) | *C. reticulata* Blanco | INRAE-CIRAD BRC |
| Granito sour orange | *C. aurantium* L. | INRAE-CIRAD BRC |
| Salustiana orange (Sweet orange) | *C. sinensis* (L.) Osb. | INRAE-CIRAD BRC |
| Chandler pomelo | *C. maxima* (Burm.) Merr. | INRAE-CIRAD BRC |
| Poncire citron | *C. medica* L. | INRAE-CIRAD BRC |
| Eureka lemon | *C. limon* (L.) Burm. | INRAE-CIRAD BRC |
| Mexican lime | *C. aurantifolia* (Christm.) Swing. | INRAE-CIRAD BRC |
| Sweet lime | *C. aurantifolia* (Christm.) Swing. | INRAE-CIRAD BRC |
| Pursha lime | *C. aurantifolia* (Christm.) Swing. | INRAE-CIRAD BRC |

1. * Corresponding author at Department of Food Science and Technology, National University of Singapore, S14 Level 5, Science Drive 2, Singapore 117542; Mane SEA PTE LTD, 3 Biopolis Drive, #07-17/18/19 Synapse, Singapore 138623

   E-mail address: [gsgpbiy@gmail.com](mailto:gsgpbiy@gmail.com) (B.Yu); [fstlsq@nus.edu.sg](mailto:fstlsq@nus.edu.sg) (S.Q. Liu) [↑](#footnote-ref-1)
