# Supplementary material for: Distinguishing citrus varieties based on genetic and compositional analyses
Source: PLoS One. 2022 Apr 18;17(4):e0267007. doi: 10.1371/journal.pone.0267007 (PMC9015143; doi:10.1371/journal.pone.0267007)
Supplement: S2 Table — (a) Descriptions of the 16 SSR markers used. (b) Allelic sizes of the amplified DNA fragments at the 16 loci of SSR for the studied citrus genotypes. (ZIP) [file pone.0267007.s002.zip › SupplementaryTable_S2(b).docx]

**Distinguishing citrus varieties based on genetic and compositional analyses**

**Rui Min Vivian Goh^a^, Aileen Pua^a,b^, Francois Luro^c^, Kim Huey Ee^b^, Yunle Huang^a,b^, Elodie Marchi^c^, Shao Quan Liu^a*^, Benjamin Lassabliere^b^, Bin Yu^b^^[[1]](#footnote-1)^***

^a^Department of Food Science and Technology, National University of Singapore, S14 Level 5, Science Drive 2, Singapore 117542

^b^Mane SEA PTE LTD, 3 Biopolis Drive, #07-17/18/19 Synapse, Singapore 138623

^c^UMR AGAP Institut, Univ Montpellier, CIRAD, INRAE, Institut Agro -, 20230, San Giuliano, France

Supplementary Table S2(b). Allelic sizes of the amplified DNA fragments at the 16 loci of SSR for the studied citrus genotypes

| **Citrus varieties** | **SSR markers** | | | | | | | | | | | | | | | | | | | | | | | | | | | | | | | |
| --- | --- | --- | --- | --- | --- | --- | --- | --- | --- | --- | --- | --- | --- | --- | --- | --- | --- | --- | --- | --- | --- | --- | --- | --- | --- | --- | --- | --- | --- | --- | --- | --- |
|  | 02D09 | | 01F04a | | 07D06 | | 03C08 | | 08C05 | | MEST131 | | MEST192 | | MEST488 | | MEST046 | | MEST015 | | 01C06 | | 02F07 | | 04H06 | | TAA1 | | TAA41 | | MEST088 | |
|  | all1 | all2 | all1 | all2 | all1 | all2 | all1 | all2 | all1 | all2 | all1 | all2 | all1 | all2 | all1 | all2 | all1 | all2 | all1 | all2 | all1 | all2 | all1 | all2 | all1 | all2 | all1 | all2 | all1 | all2 | all1 | all2 |
| Sunki | 253 | 255 | 231 | 238 | 212 | 214 | 235 | 235 | 195 | 199 | 153 | 171 | 239 | 239 | 145 | 151 | 239 | 239 | 212 | 212 | 150 | 152 | 187 | 189 | 210 | 210 | 180 | 180 | 174 | 174 | 126 | 132 |
| Ponka | 255 | 257 | 214 | 216 | 206 | 214 | 232 | 233 | 171 | 175 | 165 | 171 | 233 | 247 | 149 | 157 | 236 | 263 | 209 | 212 | 152 | 184 | 189 | 189 | 208 | 210 | 180 | 182 | 171 | 172 | 132 | 138 |
| Cleopatra | 245 | 247 | 216 | 238 | 206 | 206 | 246 | 248 | 185 | 187 | 177 | 177 | 239 | 239 | 145 | 161 | 239 | 239 | 209 | 212 | 150 | 150 | 189 | 189 | 210 | 216 | 180 | 180 | 165 | 180 | 132 | 150 |
| Willow leaf | 253 | 255 | 214 | 220 | 196 | 206 | 233 | 251 | 183 | 193 | 165 | 171 | 239 | 239 | 145 | 149 | 239 | 239 | 209 | 215 | 152 | 184 | 189 | 189 | 210 | 216 | 180 | 182 | 165 | 165 | 132 | 132 |
| Sour orange | 253 | 253 | 208 | 216 | 182 | 214 | 240 | 246 | 175 | 181 | 166 | 171 | 239 | 259 | 145 | 153 | 239 | 245 | 198 | 212 | 150 | 178 | 189 | 189 | 204 | 210 | 180 | 188 | 152 | 163 | 120 | 132 |
| Sweet orange | 247 | 247 | 204 | 214 | 184 | 206 | 232 | 237 | 171 | 191 | 165 | 171 | 239 | 243 | 145 | 159 | 236 | 263 | 198 | 206 | 152 | 178 | 189 | 203 | 204 | 210 | 180 | 182 | 156 | 172 | 120 | 138 |
| Chandler pomelo | 243 | 243 | 208 | 216 | 182 | 184 | 237 | 237 | 161 | 161 | 165 | 165 | 219 | 241 | 145 | 145 | 248 | 263 | 198 | 198 | 168 | 178 | 201 | 203 | 204 | 204 | 182 | 186 | 146 | 147 | 120 | 120 |
| Poncire citron | 249 | 249 | 204 | 204 | 186 | 190 | 235 | 235 | 148 | 171 | 158 | 158 | 228 | 228 | 139 | 139 | 242 | 242 | 206 | 206 | 183 | 188 | 212 | 212 | 206 | 208 | 198 | 198 | 152 | 161 | 120 | 120 |
| Eureka lemon | 251 | 253 | 204 | 216 | 182 | 186 | 235 | 240 | 171 | 181 | 158 | 171 | 228 | 239 | 139 | 153 | 239 | 242 | 198 | 206 | 178 | 188 | 189 | 212 | 206 | 210 | 188 | 198 | 163 | 168 | 120 | 120 |
| Mexican lime | 249 | 265 | 192 | 204 | 186 | 190 | 224 | 267 | 154 | 169 | 147 | 164 | 228 | 243 | 139 | 147 | 236 | 242 | 206 | 206 | 167 | 190 | 197 | 212 | 206 | 211 | 186 | 198 | 152 | 187 | 120 | 120 |
| Sweet lime | 251 | 257 | 204 | 214 | 190 | 212 | 243 | 249 | 171 | 181 | 153 | 158 | 228 | 247 | 139 | 157 | 236 | 242 | 198 | 206 | 150 | 190 | 203 | 212 | 208 | 208 | 180 | 198 | 152 | 160 | 120 | 132 |
| Pursha lime | 253 | 253 | 198 | 216 | 206 | 206 | 240 | 265 | 181 | 199 | 159 | 165 | 233 | 237 | 139 | 139 | 239 | 242 | 206 | 215 | 154 | 183 | 189 | 205 | 210 | 216 | 180 | 198 | 183 | 199 | 126 | 126 |
| Dalandan | 247 | 255 | 214 | 238 | 196 | 212 | 233 | 251 | 193 | 193 | 165 | 171 | 239 | 255 | 145 | 161 | 239 | 239 | 209 | 215 | 150 | 152 | 189 | 189 | 208 | 210 | 180 | 180 | 165 | 165 | 120 | 138 |
| Pontianak | 247 | 253 | 214 | 224 | 214 | 214 | 232 | 251 | 183 | 183 | 165 | 165 | 231 | 233 | 149 | 157 | 236 | 263 | 209 | 212 | 150 | 184 | 187 | 203 | 208 | 210 | 180 | 182 | 148 | 176 | 120 | 126 |
| Qicheng | 247 | 255 | 204 | 214 | 184 | 206 | 232 | 237 | 171 | 191 | 165 | 171 | 239 | 243 | 145 | 159 | 236 | 263 | 198 | 206 | 152 | 178 | 189 | 203 | 204 | 210 | 180 | 182 | 156 | 172 | 120 | 138 |
| Nagpur | 255 | 257 | 214 | 216 | 206 | 214 | 232 | 233 | 177 | 177 | 165 | 171 | 233 | 245 | 149 | 157 | ND | ND | 209 | 212 | 152 | 184 | 189 | 189 | 208 | 210 | 180 | 182 | 172 | 172 | 132 | 138 |
| Mosambi | 247 | 247 | 204 | 214 | 184 | 206 | 232 | 237 | 171 | 191 | 165 | 171 | 239 | 243 | 145 | 159 | 236 | 263 | 198 | 206 | 152 | 178 | 189 | 203 | 204 | 210 | 180 | 182 | 156 | 172 | 120 | 138 |
| Ma Nao Pan | 249 | 265 | 192 | 204 | 186 | 190 | 224 | 267 | 154 | 169 | 147 | 164 | 228 | 243 | 139 | 147 | 236 | 242 | 206 | 206 | 167 | 190 | 197 | 212 | 206 | 211 | 186 | 198 | 152 | 187 | 120 | 120 |
| Chanh Giay | 249 | 265 | 192 | 204 | 186 | 190 | 224 | 267 | 154 | 169 | 147 | 164 | 228 | 243 | 139 | 147 | 236 | 242 | 206 | 206 | 167 | 190 | 197 | 212 | 206 | 211 | 186 | 198 | 152 | 187 | 120 | 120 |

1. * Corresponding author at Department of Food Science and Technology, National University of Singapore, S14 Level 5, Science Drive 2, Singapore 117542; Mane SEA PTE LTD, 3 Biopolis Drive, #07-17/18/19 Synapse, Singapore 138623

   E-mail address: [gsgpbiy@gmail.com](mailto:gsgpbiy@gmail.com) (B.Yu); [fstlsq@nus.edu.sg](mailto:fstlsq@nus.edu.sg) (S.Q. Liu) [↑](#footnote-ref-1)
