# Supplementary material for: Distinguishing citrus varieties based on genetic and compositional analyses
Source: PLoS One. 2022 Apr 18;17(4):e0267007. doi: 10.1371/journal.pone.0267007 (PMC9015143; doi:10.1371/journal.pone.0267007)
Supplement: S2 Table — (a) Descriptions of the 16 SSR markers used. (b) Allelic sizes of the amplified DNA fragments at the 16 loci of SSR for the studied citrus genotypes. (ZIP) [file pone.0267007.s002.zip › SupplementaryTable_S2(a).docx]

**Distinguishing citrus varieties based on genetic and compositional analyses**

**Rui Min Vivian Goh^a^, Aileen Pua^a,b^, Francois Luro^c^, Kim Huey Ee^b^, Yunle Huang^a,b^, Elodie Marchi^c^, Shao Quan Liu^a*^, Benjamin Lassabliere^b^, Bin Yu^b^^[[1]](#footnote-1)^***

^a^Department of Food Science and Technology, National University of Singapore, S14 Level 5, Science Drive 2, Singapore 117542

^b^Mane SEA PTE LTD, 3 Biopolis Drive, #07-17/18/19 Synapse, Singapore 138623

^c^UMR AGAP Institut, Univ Montpellier, CIRAD, INRAE, Institut Agro -, 20230, San Giuliano, France

Supplementary Table S2(a). Descriptions of the 16 SSR markers used

| **Locus Id** | **SSR type** | **Scaffold** | **Position (NT)** | **Forward primer sequence** | **Reverse primer sequence** | **AT^a^ (°C)** | **Size range^b^** | **Reference** |
| --- | --- | --- | --- | --- | --- | --- | --- | --- |
| Ci02D09 | (TC) | 2 | 5 821 030 | AATGATGAGGGTAAAGATG | ACCCATCACAAAACAGA | 55 | 241-265 | Cuenca et al. 2011 |
| Ci04H06 | (AG) | 2 | 8 097 528 | CAAAGTGGTGAAACCTG | GGACATAGTGAGAAGTTGG | 55 | 206-217 | Cuenca et al. 2011 |
| Ci03C08 | (TC) | 2 | 27 340 105 | GCTTCTTACATTCCTCAAA | CAGAGACAGCCAAGAGA | 55 | 223-285 | Cuenca et al. 2011 |
| Mest046 | (CAA) | 2 | 33 532 441 | GGTGAGCATCTGGACGACTT | GAACCAGAATCAGAACCCGA | 55 | 230-256 | Demarq et al. 2021 |
| TAA41 | (TAA) | 2 | 35 861 194 | ACATGCAGTGCTATAATGAATG | AGGTCTACATTGGCATTGTC | 55 | 127-162 | Kijas et al. 1997 |
| Mest131 | (GCCCCA) | 3 | 50 550 703 | GCTGTCACGTTGGGTGTATG | TACCTCCACGTGTCAAACCA | 55 | 147-177 | Demarq eat al. 2021 |
| Ci07D06 | (TC)/(AC) | 4 | 7 293 957 | TCAATTCCTCTAGTGTGTGT | CCTTTTCACAGTTTGCTAT | 55 | 182-214 | Cuenca et al. 2011 |
| Mest088 | (GA) | 5 | 36 034 967 | ATGAGAGCCAAGAGCACGAT | GCCTGTTTGCTTTCTCTTTCTC | 55 | 99-130 | Demarq eat al. 2021 |
| Ci01C06 | (TC)N(TC) | 6 | 24 791 029 | TGGAGACACAAAGAAGAA | GGACCACAACAAAGACAG | 50 | 131-170 | Cuenca et al. 2011 |
| Mest192 | (AT) | 6 | 17 474 923 | CTTGGCACCATCAACACATC | CGCGGATCATCTAGCATACA | 55 | 200-240 | Aleza et al 2011 |
| Mest488 | (CT) | 6 | 21 253 842 | CTTTGCGTGTTTGTGCTGTT | CACGCTCTTGACTTTCTCCC | 55 | 135-165 | Demarq eat al. 2021 |
| TAA1 | (TAA) | 6 | 24 896 288 | AAGAAGAAGAGCCCCCATTAGC | GACAACATCAACAACAGCAAGAGC | 55 | 161-180 | Kijas et al. 1997 |
| Ci01F04a | (CT)N(CAG) | 8 | 1 063 777 | TGCTGCTGCTGTTGTTGTTCT | AAGCATTTAGGGAGGGTCACT | 55 | 190-228 | Froelicher et al. 2008 |
| Ci02F07 | (AC) | 8 | 15 057 658 | TGCTGGTTTTCAGATACTT | GCAGCGTTTGTTTTCT | 55 | 187-229 | Froelicher et al. 2008 |
| Mest015 | (GAG) | 8 | 24 850 644 | GCCTCGCATTCTCTTGACTC | TTATTACGAAGCGGAGGTGG | 55 | 198-215 | Demarq et al. 2021 |
| Ci08C05 | (GA) | 9 | 25 537 858 | TCCACAGATTGCCCATTA | CCCTAAAAACCAAGTGACA | 55 | 126-180 | Froelicher et al. 2008 |

^a^Annealing temperature for PCR.
^b^Range of amplified fragment sizes in nucleotides.

1. * Corresponding author at Department of Food Science and Technology, National University of Singapore, S14 Level 5, Science Drive 2, Singapore 117542; Mane SEA PTE LTD, 3 Biopolis Drive, #07-17/18/19 Synapse, Singapore 138623

   E-mail address: [gsgpbiy@gmail.com](mailto:gsgpbiy@gmail.com) (B.Yu); [fstlsq@nus.edu.sg](mailto:fstlsq@nus.edu.sg) (S.Q. Liu) [↑](#footnote-ref-1)
