# Supplementary material for: Distinguishing citrus varieties based on genetic and compositional analyses
Source: PLoS One. 2022 Apr 18;17(4):e0267007. doi: 10.1371/journal.pone.0267007 (PMC9015143; doi:10.1371/journal.pone.0267007)
Supplement: S3 Table — (DOCX) [file pone.0267007.s003.docx]

**Distinguishing citrus varieties based on genetic and compositional analyses**

**Rui Min Vivian Goh^a^, Aileen Pua^a,b^, Francois Luro^c^, Kim Huey Ee^b^, Yunle Huang^a,b^, Elodie Marchi^c^, Shao Quan Liu^a*^, Benjamin Lassabliere^b^, Bin Yu^b^^[[1]](#footnote-1)^***

^a^Department of Food Science and Technology, National University of Singapore, S14 Level 5, Science Drive 2, Singapore 117542

^b^Mane SEA PTE LTD, 3 Biopolis Drive, #07-17/18/19 Synapse, Singapore 138623

^c^UMR AGAP Institut, Univ Montpellier, CIRAD, INRAE, Institut Agro -, 20230, San Giuliano, France

Supplementary Table S3. Chemical properties of compounds in Tables 1(a) and 1(b) used for identification

| **Compound** | **Formula** | **Adduct** | **m/z** | **RT (min)** | **MS/MS fragments** |
| --- | --- | --- | --- | --- | --- |
| 7-Methoxycoumarin | C_10_H­_8_O_3_ | [M+H]^+^ | 177.0545 | 13.351 | 78.0461^a^ |
| Bergamotine | C_21_H_22_O_4_ | [M+H]^+^ | 339.1590 | 21.215 | 147.0431^a^ |
| Bergapten | C_12_H_8_O_4_ | [M+H]^+^ | 217.0495 | 15.631 | 90.0461^a^ |
| Chrysoeriol-7-glucoside | C_22_H_22_O_11_ | [M-H]^-^ | 461.1089 | 11.630 | 255.0297^b^ |
| Citropten | C_11_H_10_O_4_ | [M+H]^+^ | 207.0651 | 15.386 | 65.0384^b^ |
| Didymin | C_28_H_34_O_14_ | [M+H]^+^ | 595.2021 | 13.978 | 153.0176^b^ |
| Eriocitrin | C_27_H_32_O_15_ | [M-H]^-^ | 595.1668 | 9.425 | 151.0035^a^ |
| Hesperidin | C_28_H_34_O_15_ | [M-H]^-^ | 609.1825 | 11.524 | 301.0725^a^ |
| Homoeriodicytol-7-glucoside | C_22_H_24_O_11_ | [M+H]^+^ | 465.1391 | 11.544 | 151.0038^a^ |
| Hyperoside | C_21_H_20_O_12_ | [M-H]^-^ | 463.0882 | 9.518 | 271.0250^b^ |
| Isomangiferin | C_19_H_18_O_11_ | [M+H]^+^ | 423.0921 | 7.291 | 273.0383^a^ |
| Isoquercetin | C_21_H_20_O_12_ | [M-H]^-^ | 463.0882 | 9.729 | 303.0488^a^ |
| Isorhamnetin | C_16_H_12_O_7_ | [M-H]^-^ | 315.0510 | 15.138 | 63.0247^b^ |
| Isorhamnetin-3-glucoside | C_22_H_22_O_12_ | [M-H]^-^ | 477.1038 | 11.138 | 243.0298^b^ |
| Isorhamnetin-3-neohesperidoside | C_28_H_32_O_16_ | [M-H]^-^ | 623.1617 | 9.929 | 299.0200^b^ |
| Isorhamnetin-3-rutinoside | C_28_H_32_O_16_ | [M-H]^-^ | 623.1617 | 10.795 | 299.0197^b^ |
| Isorhoifolin | C_27_H_30_O_14_ | [M-H]^-^ | 577.1563 | 10.803 | 269.0463^a^ |
| Isoscoparin (Chrysoeriol-6-glucoside) | C_22_H_22_O_11_ | [M-H]^-^ | 461.1089 | 10.089 | 299.0521^a^ |
| Isovitexin | C_21_H_20_O_10_ | [M+H]^+^ | 433.1128 | 9.537 | 283.0593^a^ |
| Kaempferol-3-neohesperidoside | C_27_H_30_O_15_ | [M-H]^-^ | 593.1512 | 9.665 | 255.0300^b^ |
| Kaempferol-3-rutinoside | C_27_H_30_O_15_ | [M-H]^-^ | 593.1512 | 10.502 | 255.0304^b^ |
| Limonin | C_26_H_30_O_8_ | [M+H]^+^ | 471.2013 | 16.300 | 161.0591^a^ |
| Luteolin-6-glucoside | C_21_H_20_O_11_ | [M-H]^-^ | 447.0933 | 8.417 | 133.0293^b^ |
| Mangiferin | C_19_H_18_O_11_ | [M+H]^+^ | 423.0921 | 6.998 | 273.0343^a^ |
| Narirutin | C_27_H_32_O_14_ | [M-H]^-^ | 579.1719 | 10.626 | 151.0035^b^ |
| Neohesperidin | C_28_H_34_O_15_ | [M-H]^-^ | 609.1825 | 11.947 | 301.0719^a^ |
| Nobiletin | C_21_H_22_O_8_ | [M+H]^+^ | 403.1387 | 16.798 | 183.0287^b^ |
| Nomilin | C_28_H_34_O_9_ | [M+H]^+^ | 515.2275 | 16.873 | 161.0593^a^ |
| Obacunone | C_26_H_30_O_7_ | [M-H]^-^ | 453.1919 | 17.470 | 315.0990^a^ |
| Orientin (Luteolin-8-glucoside) | C_21_H_20_O_11_ | [M-H]^-^ | 447.0933 | 8.600 | 133.0294^b^ |
| Prunin | C_21_H_22_O_10_ | [M-H]^-^ | 433.1140 | 11.040 | 151.0038^a^ |
| Quercetin-3-neohesperidoside | C_27_H_30_O_16_ | [M-H]^-^ | 609.1461 | 8.838 | 271.0257^b^ |
| Rhoifolin | C_27_H_30_O_14_ | [M-H]^-^ | 577.1563 | 11.194 | 269.0455^a^ |
| Rutin | C_27_H_30_O_16_ | [M-H]^-^ | 609.1461 | 9.442 | 301.0346^a^ |
| Scoparone | C_11_H_10_O_4_ | [M+H]^+^ | 207.0651 | 11.492 | 107.0488^a^ |
| Sinensetin | C_20_H_20_O_7_ | [M+H]^+^ | 373.1281 | 16.188 | 153.0180^b^ |
| Tangeretin | C_20_H_20_O_7_ | [M+H]^+^ | 373.1281 | 17.406 | 183.0286^b^ |
| Vicenin-1 | C_26_H_28_O_14_ | [M-H]^-^ | 563.1406 | 8.51 | 353.0661^a^ |
| Vicenin-2 | C_27_H_30_O_15_ | [M-H]^-^ | 593.1512 | 7.52 | 353.0671^a^ |
| Vitexin | C_21_H_20_O_10_ | [M+H]^+^ | 433.1128 | 9.441 | 283.0592^b^ |

^a^Major fragment at CE 40 V.
^b^Major fragment at CE 60 V.

1. * Corresponding author at Department of Food Science and Technology, National University of Singapore, S14 Level 5, Science Drive 2, Singapore 117542; Mane SEA PTE LTD, 3 Biopolis Drive, #07-17/18/19 Synapse, Singapore 138623

   E-mail address: [gsgpbiy@gmail.com](mailto:gsgpbiy@gmail.com) (B.Yu); [fstlsq@nus.edu.sg](mailto:fstlsq@nus.edu.sg) (S.Q. Liu) [↑](#footnote-ref-1)
